# An Investigation on Substitution of Ag Atoms for Al or Ti Atoms in the Ti_2_AlC MAX Phase Ceramic Based on First-Principles Calculations

**DOI:** 10.3390/ma14227068

**Published:** 2021-11-21

**Authors:** Guochao Wang, Jiahe Zhou, Weijian Chen, Jianguo Yang, Jie Zhang, Yanming He

**Affiliations:** 1 38th Research Institute, China Electronics Technology Group Corporation, Hefei 230000, China; hitwgc@gmail.com; 2 Institute of Process Equipment and Control Engineering, Zhejiang University of Technology, Hangzhou 310014, China; zhoujiahe@zjut.edu.cn (J.Z.); weijianchen@zjut.edu.cn (W.C.); yangjg@zjut.edu.cn (J.Y.); 3 School of Materials Science and Engineering, Harbin Institute of Technology, Harbin 150001, China

**Keywords:** Ti_2_AlC MAX phase ceramic, first-principles calculation, substitution behavior, bonding characteristic, stability

## Abstract

The present work introduced first-principles calculation to explore the substitution behavior of Ag atoms for Al or Ti atoms in the Ti_2_AlC MAX phase ceramic. The effect of Ag substitution on supercell parameter, bonding characteristic, and stability of the Ti_2_AlC was investigated. The results show that for the substitution of Ag for Al, the Al-Ti bond was replaced by a weaker Ti-Ag bond, decreasing the stability of the Ti_2_AlC. However, the electrical conductivity of the Ti_2_AlC was enhanced after the substitution because of the contribution of Ag 4*d* orbital electrons toward the density of states (DOS) at the Fermi level coupled with the filling of Ti *d* orbital electrons. For the substitution of Ag for Ti, new bonds, such as Ag-Al bond, Ag-C bond, Al-Al bond, Ti-Ti anti-bond, and C-C anti-bond were generated in the Ti_2_AlC. The Ti-Ti anti-bond was strengthened as well as the number of C-C anti-bond was increased with increasing the substitution ratio of Ag for Ti. Similar to the substitution of Ag for Al, the stability of the Ti_2_AlC also decreased because the original Al-Ti bond became weaker as well as the Ti-Ti and C-C anti-bonds were generated during the substitution of Ag for Ti. Comparing with the loss of Ti *d* orbital electrons, Ag 4*d* orbits contributed more electrons to the DOS at the Fermi level, improving the electrical conductivity of the Ti_2_AlC after substitution. Based on the calculation, the substitution limit of Ag for Al or Ti was determined. At last, the substitution behavior of Ag for Al or Ti was compared to discriminate that Ag atoms would tend to preferentially substitute for Ti atoms in Ti_2_AlC. The current work provides a new perspective to understand intrinsic structural characteristic and lattice stability of the Ti_2_AlC MAX phase ceramic.

## 1. Introduction

The MAX phases, a group of ternary compounds with layered nanostructure, have attracted interest in the field of ceramics in recent 20 years. They have the general formula of M*_n_*_+1_AX*_n_* (*n* = 1, 2 or 3), where M belongs to a transition metal, A is an element of group IIIA or VIA, and X pertains to C or N [1,2,3]. When *n* = 1, they are named as 211 phases with the typical representative of Ti_2_AlC. The Ti_2_AlC has a low density, high Young’s modulus, good thermal/electrical conductivity, superior oxidation resistance, and self-lubricating capability, making them ideal structural materials in many fields, particularly in high-temperature occasions [4,5,6,7,8,9,10].

It should be mentioned that Ti, Al, and C atoms in the Ti_2_AlC lattices can be substituted by other atoms [11,12,13,14,15,16,17,18,19]. Through the substitution, the Ti_2_AlC modified will exhibit distinctive properties to accommodate the requirements in diverse environments, broadening their applications [20,21,22]. F. Meng et al. [11] proposed the Ti_2_AC could be strengthened through substituting Ti with V. Compared to the Ti_2_AC, the Vickers hardness, flexural, and shear strength of the (Ti_0.8_,V_0.2_)_2_AlC were enhanced by 29%, 36%, and 45%, respectively. J. Rosen et al. [12] deposited Ti_2_AlC film with the aid of a multiple cathode pulsed cathodic arc and found that a substantial amount of oxygen was incorporated into the lattice. The first-principles calculation described that oxygen atoms could substitute for carbon atoms and then tailor properties of the Ti_2_AlC. C.J. Lu et al. [13] observed that the presence of Si element made the lattice constants of Ti_2_AlC shrink in the Ti_2_AlC brazed joints using transmission electron microscope, proving the Ti_2_(Al_1−*x*_,Si*_x_*)C structure was stable in the vibration aspect through first-principles calculation. We once adopted pure Ag to braze the Ti_2_AlC at 1030 °C, and found that Al and Ti migrated out of the Ti_2_AlC while Ag diffused into the lattices along the vacancy channels driven by the migration of Al and Ti [23]. The high-resolution transmission electron microscope analysis further demonstrated that Al and Ti atoms could be replaced by Ag atoms in the Ti_2_AlC [24]. However, the effect of substitution behavior of Ag on the lattice structure and thermodynamic stability of the Ti_2_AlC remained unknown through the experimental investigation. Based on this, the first-principles calculation based on density functional theory (DFT) was carried out in the present work to explore the effect of Ag substitution behavior and substitution degree on supercell parameter, electronic structure, bonding characteristic, and stability of the Ti_2_AlC. The changes in bonding characteristic and charge distribution between the atoms were then acquired for the Ti_2_AlC during the interaction. Based on this the interaction mechanism between Ag and Ti_2_AlC were illuminated, which will provide a new perspective to understand the intrinsic structural characteristic and lattice stability of the Ti_2_AlC MAX phase ceramics.

## 2. Materials and Methods

**Figure 1:**
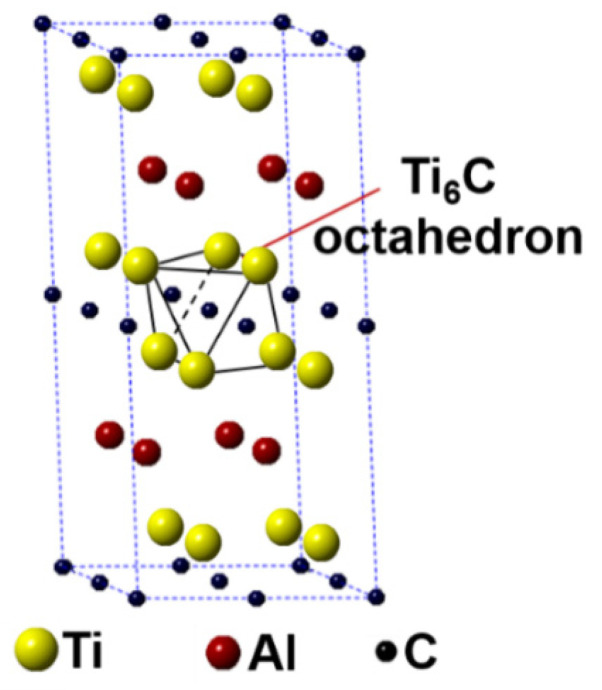
Crystal structure of Ti_2_AlC supercell (2 × 2 × 1 unit cells).

In this work, the first-principles calculation was conducted using the Cambridge Sequential Total Energy Package (CASTEP) in the Materials Studio [25,26]. The Ti_2_AlC unit cell having 8 atoms crystallizes with a hexagonal structure having the *P*6_3_/*mmc* space group (Ti at 4*f*, Al at 2*c* and C at 2*a* Wyckoff positions). A 2 × 2 × 1 Ti_2_AlC supercell containing 32 atoms (16 Ti atoms, 8 Al atoms, and 8 C atoms) was built to model the substitution behavior, as shown in Figure 1. Such a supercell size can ideally reflect the structural characteristics of Ti_2_AlC [13,27]. The Ti_2_AlC consists of one Al atomic layer and one Ti_6_C octahedral layer alternately distributed, in where C atoms are located in the octahedral gap formed by Ti atoms. For the original Ti_2_AlC, Ti_2_(Al_1−*x*_,Ag*_x_*)C, and (Ti_1−*y*_,Ag*_y_*)_2_AlC, the equilibrium lattice parameter and ground-state electronic structure were calculated by adjusting the substitution ratio of Ag for Al and Ti in the supercell. J.Y. Wang et al. [27] reported that the Ti_2_AlC was destabilized when the vacancy concentration of Al exceeded 50% in the lattice. It should be mentioned that the generation of Al vacancies was prerequisite for the replacement of Ag for Al in the Ti_2_AlC. For this the substitution ratio (*x*) of Ag for Al was set as 0.125, 0.25, 0.375, and 0.5, and accordingly the number of Al atoms substituted was 1, 2, 3, and 4, respectively. The (Ti_0.8125_,Ag_0.1875_)_2_AlC supercell (Ti atoms substituted: 3) cannot be converged during optimization, suggesting the structure with more than 3 substituted Ti atoms (*y* > 0.125) cannot exist stably. So, the substitution ratio (*y*) of Ag for Ti was set as 0.0625 and 0.125, and accordingly the number of Ti atoms substituted was 1 and 2, respectively. For the lattice with substituted atoms over two, the effect of substitution position on supercell structure should be took into consideration, conforming to the principle of total energy minimization. The Vanderbilt ultrasoft pseudopotential was employed to describe the interaction between electrons and ions during the calculation while the exchange-correlation energy function was constructed by the generalized gradient approximation (GGA-PW91) [28]. The Monkhorst-Pack grids were used to configure the *k*-space within the Brillouin zone [29], and the *k*-point mesh was set as 10 × 10 × 2, along with a plane-wave cutoff energy of 450 eV. Finally, the convergence accuracy of geometrical optimization was chosen to be ultra-fine. These parameters used were sufficient to give rise to well converged total energy and geometrical configuration [30,31,32,33].

## 3. Results and Discussion

### 3.1. Crystal Structure and Stability of the Ti_2_(Al_1−*x*_, Ag_*x*_)C

**Figure 2:**
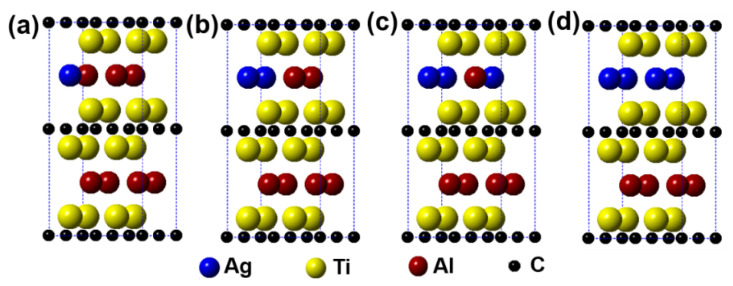
Models of the Ti_2_(Al_1−*x*_,Ag_*x*_)C supercells with different substitution ratios of Ag for Al: (**a**) Ti_2_(Al_0.875_,Ag_0.125_)C; (**b**) Ti_2_(Al_0.75_,Ag_0.25_)C; (**c**) Ti_2_(Al_0.625_,Ag_0.375_)C; (**d**) Ti_2_(Al_0.5_,Ag_0.5_)C.

Figure 2 describes the models of Ti_2_(Al_1−*x*_,Ag*_x_*)C (*x* = 0.125, 0.25, 0.375, 0.5) supercells, in which the number of Al substituted is 1, 2, 3, and 4, respectively. It can be seen from the figure that the substitution of Ag for Al is preferentially carried out in one layer rather than random substitution. Table 1 displays the calculation results after geometrical optimization. With increasing the substitution concentration of Ag for Al in the Ti_2_(Al_1−*x*_,Ag*_x_*)C, as exhibited, the equilibrium supercell parameters of *a* and *b* show no obvious change whereas the value of *c* increases slightly, which makes the volume of supercell increase. When the substitution ratio of Ag for Al reaches 37.5%. However, the value of *c* stops growing as well as the volume of supercell decreases. Besides, it is interesting to find that as the substitution ratio increases, the parameters of *a* and *b* become different, the lattice distortion then appears. The fact suggests that the symmetry of the hexagonal Ti_2_AlC will be distorted with Ag substitution for more than 1 Al atoms. At last, it should be noted that the total energy of the system decreases with an increase in the substitution ratio of Ag for Al, whereas the formation energy and cohesive energy increase continuously, indicating that the substitution behavior of Ag for Al will decline the lattice stability of the Ti_2_AlC.

**Figure 3:**
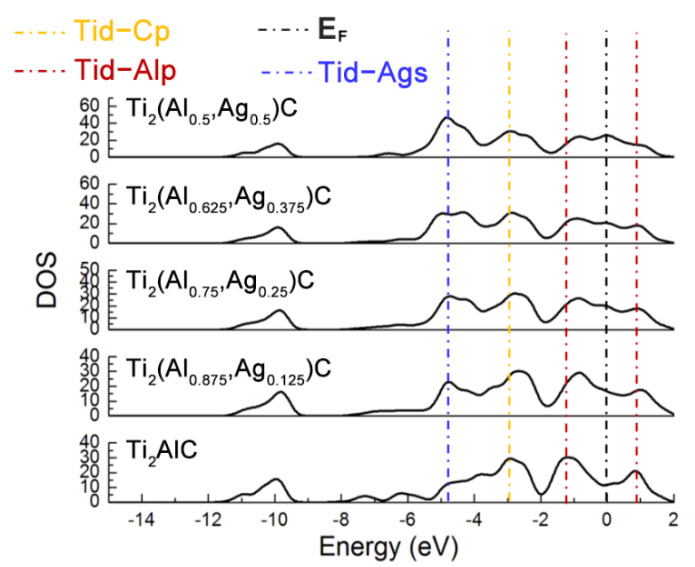
TDOS of the Ti_2_(Al_1−*x*_,Ag*_x_*)C supercells with different substitution ratios of Ag for Al.

Figure 3 displays total density of states (TDOS) for the Ti_2_(Al_1−*x*_,Ag*_x_*)C supercells with different substitution ratios of Ag for Al. The Ti *d*-C *s* hybridized peak at the energy level of −10 eV varies slightly while the hybridization effect of Ti *d*-C *p* near −2.5 eV is enhanced. The position of pseudogap triggered by Ti *d*-Al *p* hybridization near the Fermi level (*E_F_*) shifts toward a higher energy level, as well as the width of the pseudogap decreases. The fact suggests that the Ti *d*-C *s* bond is almost unaffected and stability of the Ti_2_(Al_1−*x*_,Ag*_x_*)C decreases, as the substitution proportion of Ag for Al increases. It is also noted that the TDOS of Ti_2_AlC at the *E_F_* is located at a minimum position in the curve, which is usually considered to be the critical point between the bond and anti-bond state of the structure. When the substitution proportion of Ag for Al reaches 25%, the *E_F_* is located close to one of the peaks in the TDOS, indicating that part of electrons occupy anti-bond states and the structure of Ti_2_(Al_0.75_,Ag_0.25_)C becomes unstable, being consistent with the calculated result of cohesive energy in Table 1. When the substitution proportion of Ag for Al is 37.5%, the *E_F_* is located at one of the peaks in the TDOS, indicating that such a structure is not able to arise stably. To sum up, the Ti_2_AlC remains stable with the Ag substitution ratio of 12.5% (i.e., Ti_2_(Al_0.875_,Ag_0.125_)C), whereas it becomes unstable when the substitution ratio of Ag for Al reaches 25% (i.e., Ti_2_(Al_0.75_,Ag_0.25_)C). It has been reported that the Ti_2_AlC could maintain stability with incorporation of 50% vacancy concentration of Al [27], which is much higher than the substitution limit of Ag for Al (12.5%) calculated in the current work. Therefore, it can be inferred that the substitution of Ag for Al would not only fill the Al vacancies within the Ti_2_AlC lattice but also give rise to the bonding interaction with neighboring Ti atoms.

**Figure 4:**
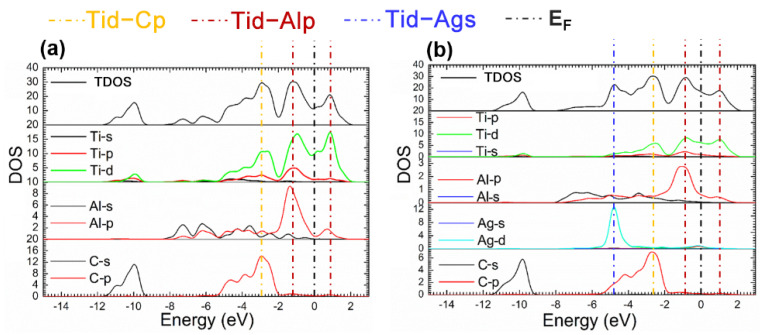
TDOS and PDOS of the Ti_2_(Al_1−*x*_,Ag_*x*_)C before and after Ag substitution: (**a**) Ti_2_AlC; (**b**) Ti_2_(Al_0.875_,Ag_0.125_)C.

The fact mentioned suggests that the Ti_2_AlC maintained structural stability without ruining its lattice with the Ag substitution ratio of 12.5%. Considering this the supercell with 12.5% Ag substitution was analyzed in the followings. Figure 4 presents the TDOS and partial density of states (PDOS) of the supercells before and after Ag substitution. It can be shown from Figure 4b that a characteristic peak can be discovered at −4.5 eV produced by 4*d* orbital electrons of Ag, and an increase in DOS is also observed at the *E_F_*, implying more filling of the *d* orbital electrons. It can be seen from the PDOS of Ag atoms that the 4*d* orbital electrons of Ag contributes to the DOS at the *E_F_*, suggesting the electrical conductivity of the Ti_2_(Al_0.875_,Ag_0.125_)C is enhanced.

**Figure 5:**
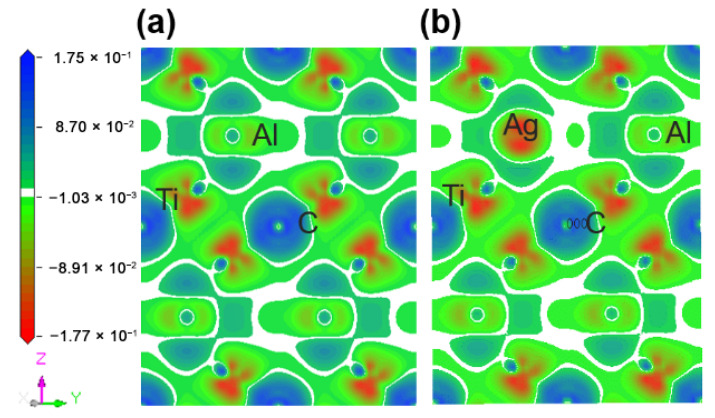
Maps of charge density difference of the Ti_2_(Al_1−*x*_,Ag_*x*_)C supercells along the crystallographic plane of $\left(10\bar{1}0\right)$: (**a**) Ti_2_AlC; (**b**) Ti_2_(Al_0.875_,Ag_0.125_)C.

Figure 5 describes the maps of charge density difference of the Ti_2_(Al_1−*x*_,Ag*_x_*)C supercells along the crystallographic plane of $\left(10\bar{1}0\right)$. Before and after the substitution of Al by Ag, the charge density difference around the substituted Al atoms was calculated to determine the bonding status and stability. It can be shown from Figure 5a that the charge density difference of C atoms is positive, showing electron acquisition, whereas Ti atoms with a negative charge density difference exhibit electron loss. In addition, both of C and Ti atoms are encapsulated inside the electron clouds. A charge accumulation can be discovered between them, and the charge density difference of Ti atoms has a strong directionality on the side close to C atom, suggesting a covalent bond between C and Ti. The charge density difference of Al atoms close to Ti atoms is positive, showing electron acquisition, but no directionality occurs between them. Similarly, there is no directionality between Ti and Ti atoms, indicating a metallic bond between them. After the substitution of Ag for Al, as presented in Figure 5b, the charge density difference of Ti and Ag decreases, implying the newly-formed Ti-Ag bond is weaker than the original Al-Ti bond.

Table 2 shows the atomic orbital charge populations of Ti_2_AlC and Ti_2_(Al_0.875_,Ag_0.125_)C supercells. For simplicity, only some atoms whose orbital electrons have been changed were listed. It should be noted that the number of extranuclear valence electrons for Ti, Al, C, and Ag are 3*s*^2^3*p*^6^3*d*^2^4*s*^2^, 3*s*^2^3*p*^1^, 2*s*^2^2*p*^2^, and 4*d*^10^5*s*^1^, respectively. It can be demonstrated from the table that electron acquisition/loss and orbital hybridization should happen during bonding of the Ti_2_AlC with electrons transferring from Ti to Al and C atoms. Particularly, C atoms have a stronger capability in gaining electrons compared to Al. Ti atoms lose part of valence electrons to become the positive charge of +0.40, while C atoms acquire electrons to become the negative charge of −0.75. A covalent bond will be produced between them. Al atoms gain a small number of valence electrons to become the negative charge of −0.05. Through examining the charge population of each atomic orbital coupled with net charge of bonding atoms, it can be inferred that the generation of Ti_2_AlC should be ascribed to 3*d* and 3*p* orbital electrons of Ti atoms, 3*p* orbital electrons of Al atoms and 2*s* and 2*p* orbital electrons of C atoms. After substitution of Ag for Al in the Ti_2_AlC, the total orbital charge population of C atoms remains almost unchanged (from 4.75 to 4.76). Ag atoms acquire electrons from adjacent Ti atoms and become the negative charge of −0.48. The increase in atomic orbital charge population of Al atoms close to or near-close to Ag atoms indicates that the hybridization between Al and Ti is weakened, being consistent with the results of DOS and charge density difference mentioned.

To clarify the changes of bond length and strength before and after the substitution of Ag for Al in the Ti_2_AlC, it is prerequisite to analyze the Mulliken bond overlap population. The higher the overlap population, the stronger the covalency of interatomic bonds will be. A strong ionic bond will be produced when the overlap population is approaching to zero. When the overlap population is zero, an ideal ionic bond emerges [34]. Table 3 lists the Mulliken bond overlap populations of the Ti_2_AlC and Ti_2_(Al_0.875_,Ag_0.125_)C supercells. In the Ti_2_AlC supercell, the overlap population and bond length of Ti-C bond are 0.39 and 2.10798 Å, respectively. The high overlap population indicates the Ti-C bond has a stronger covalency. The overlap population of Ti-Ti bond is close to zero with the bond length of 2.89219 Å, implying the adjacent Ti atoms form a metallic bond with the aid of sharing a small number of electrons. The overlap population of Al-Ti bond is 0.30, indicating a covalent bond between them. However, the Al-Ti bond has a larger bond length (2.89517 Å) as well as the charge between them has no directionality. It can be then concluded that the Al-Ti bond does not belong to a pure covalent bond, and a weak bond such as Van der Waals force occurs between them [35]. After the substitution of Ag for Al, the Ti-Ag bond will be produced. Compared to the Al-Ti bond, it can be demonstrated from Table 3 that the Ti-Ag bond has a smaller overlap population and larger bond length, suggesting that the Ti-Ag bond is weaker than the Ti-Al bond. During the transition from the Al-Ti bond to the Ti-Ag bond, the decrease in overlap population drove the electrons filling 3*d* orbits of Ti. The overlap population of the Ti-Ti bond close to the Ag atoms is therefore increased from 0.04 to 0.09.

It has been reported from Table 3 that the bond length of Ti-Ag bond was greater than Al-Ti bond, which should be responsible for the increased supercell parameter of *c* of the Ti_2_(Al_1−*x*_,Ag*_x_*)C. Similarly, the supercell parameters of *a* and *b* exhibited almost no change because the Ti-C bond was less unaffected (both of the overlap population and bond length) during the substitution. The replacement of Al-Ti bond with a weaker Ti-Ag bond should be responsible for the decline of structural stability of the Ti_2_(Al_1−*x*_,Ag*_x_*)C. In addition, it has been mentioned that electrical conductivity of the Ti_2_AlC was enhanced after the substitution, which should be ascribed to the filling of Ti *d* orbital electrons. Apart from the contribution of Ag 4*d* orbital electrons toward the electrical conductivity, the replacement of Al-Ti bond with Ti-Ag bond generated excess electrons filling the Ti *d* orbits, likewise enhancing the electrical conductivity.

### 3.2. Crystal Structure and Stability of the (Ti_1−y_, Ag_y_)_2_AlC

**Figure 6:**
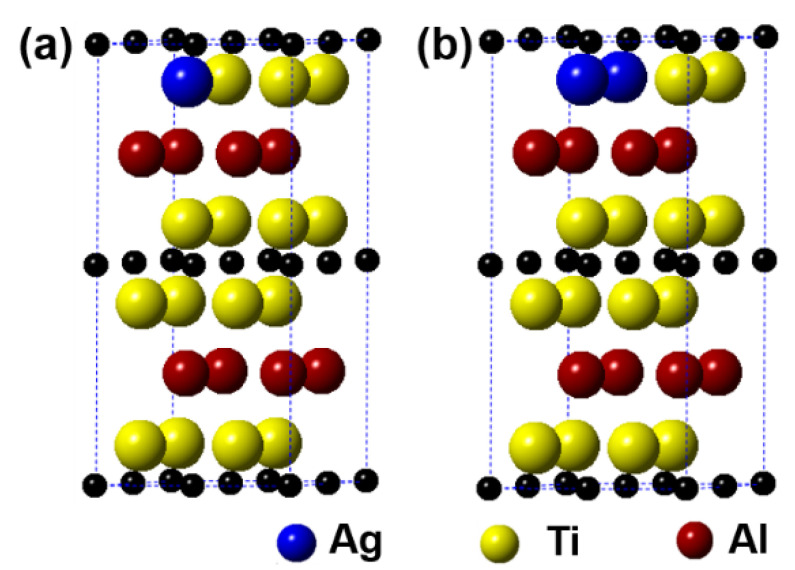
Models of the (Ti_1__−*y*_,Ag*_y_*)_2_AlC supercells with different substitution ratios of Ag for Ti: (**a**) (Ti_0.9375_,Ag_0.0625_)_2_AlC; (**b**) (Ti_0.875_,Ag_0.125_)_2_AlC.

Figure 6 displays the models of (Ti_1−*y*_,Ag*_y_*)_2_AlC (*y* = 0.0625, 0.125) supercells, in which the number of Ti substituted is 1 and 2, respectively. It can be observed from the figure that the substitution of Ag for Ti is also preferentially carried out in one Ti atomic layer, similar to the substitution behavior of Ag for Al. Table 4 presents the calculation results after geometrical optimization. With increasing the substitution ratio of Ag for Ti, the supercell parameters of *a* and *b* increase negligibly. In addition, the value of *c* first increases and then decreases, but it exhibits an upward trend. For the case of 2 Ti atoms substituted, the supercell parameters of *a* and *b* show different. Similar to the substitution of Ag for Al, the symmetry of Ti_2_AlC will also be distorted when a certain substitution ratio of Ag for Ti has been reached. The volume of supercells shows an increased trend. In addition, the total energy, formation energy, and cohesion energy increase as the Ag substitution ratio increases, indicating the decline of structural stability of the Ti_2_AlC.

**Figure 7:**
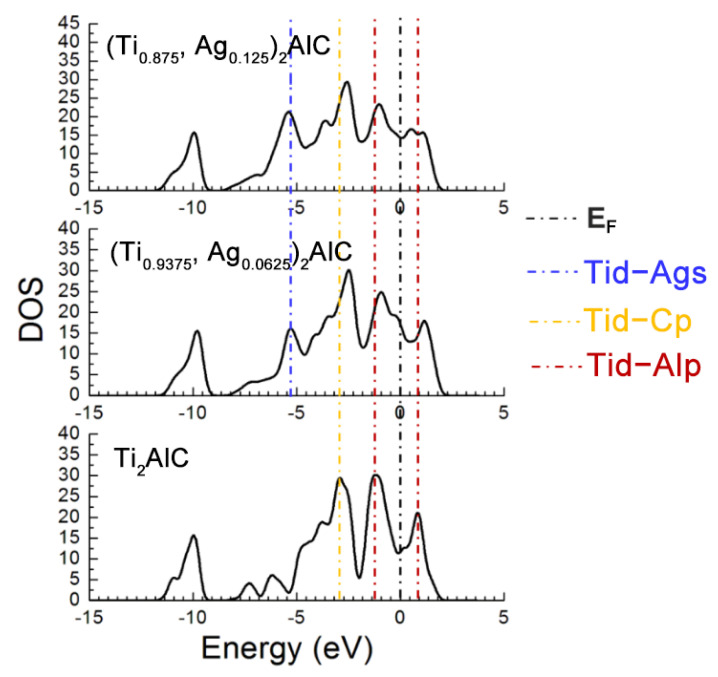
TDOS of the (Ti_1__−*y*_,Ag*_y_*)_2_AlC supercells with different substitution ratios of Ag for Ti.

Figure 7 describes the TDOS of the Ti_2_AlC and (Ti_1−*y*_,Ag*_y_*)_2_AlC supercells. As the substitution ratio of Ag for Ti increases, the height and width of Ti *d*-C *s* hybridized peak show also no change. For the Ti *d*-C *p* and Ti *d*-Ag *s* hybridized peaks, however, their height and width increase significantly. The fact indicates that the Ti *d*-C *s* bond is almost unaffected by the substitution, whereas the Ti *d*-C *p* hybridization is enhanced with increasing the substitution ratio of Ag for Ti. The position of pseudogap formed by Ti *d*-Al *p* hybridization deviates from *E_F_*, indicating the decrease in structural stability, which is consistent with the calculation results of formation and cohesive energies listed in Table 4. With increasing the substitution ratio of Ag for Ti, the DOS at *E_F_* first increases and then remains constant, which should be ascribed to the substitution of Ag for Ti weakens the contribution of Ti *d* orbital electrons toward the DOS at *E_F_*. It should be mentioned that the contribution of Ag 4*d* orbital electrons toward the DOS at *E_F_* is greater than the loss of Ti *d* orbital electrons. Therefore, the electronic conductivity of (Ti_1−*y*_,Ag*_y_*)_2_AlC supercells will also be improved, compared to the Ti_2_AlC.

Table 5 lists the Mulliken bond overlap populations of the (Ti_1−*y*_, Ag*_y_*)_2_AlC supercells. For the (Ti_0.9375_,Ag_0.0625_)_2_AlC, the over population of the Ti-C bond increases from 0.39 to 0.44, implying an increase in the strength of the covalent bond. It can also be seen that the overlap population of the Al-Ti bond decreases, meaning a decrease in bond strength. The overlap population of part of the Ti-Ti bonds is −0.03, belonging to an anti-bond state. The transformation from a positive bond state to an anti-bond state for the Ti-Ti bond indicates the decline in structural stability of the (Ti_0.9375_,Ag_0.0625_)_2_AlC. Besides that, part of the C-C bonds with an anti-bond state has the overlap population of −0.09, which is also adverse to the structural stability. It is interesting to note that Ag atoms are hybridized with Al and C atoms in the structure. Similar to the Al-Ti bond, the overlap population and bond length of the Ag-Al bond is calculated to be 0.33 and 2.69190 Å, respectively. The Ag-C bond has the overlap population of 0.20 and bond length of 2.46208 Å, which is weaker than the Ti-C bond. The emergence of Ag-Al and Ag-C bonds indicates that Ag as a transition element substituted for Ti in the Ti_2_AlC, and produced the bonding with Al and C atom, respectively. It should be noted that the supercell parameters *a* and *b* in the Ti_2_AlC would be governed by the Ti-C bond [36]. After substitution, the bond length of Ag-C (2.46208 Å) was greater than Ti-C (2.08314 Å). At the same time, the combination of Ti-Ti and C-C anti-bonds made the bond length increase further. Therefore, the supercell parameters of *a* and *b* increased. The Al-Ti bond was the key factor in determining the supercell parameter of *c* of the Ti_2_AlC. The Ag-Al bond in (Ti_0.9375_,Ag_0.0625_)_2_AlC and Al-Ti bond in Ti_2_AlC had similar bond strength and length, which further demonstrates that the substitution of Ag for Ti had a negligible impact on the supercell parameter of *c*, being consistent with the calculated results of the supercell parameters listed in Table 4.

In the (Ti_0.875_,Ag_0.125_)_2_AlC) structure near to the Ag atoms, the Ti-C bond hybridization is enhanced with increasing the substitution ratio of Ag for Ti, whereas the Al-Ti bond hybridization is weakened. The Ag-C bond, Ag-Al bond, and Ti-Ti anti-bond are strengthened. The C-C anti-bond is not strengthened, but its number increases. It is interesting to discover that the Al-Al bond happens in the (Ti_0.875_,Ag_0.125_)_2_AlC with the population of 0.23 and bond length of 2.96005 Å, but it does not appear in the original Ti_2_AlC. The charge transfer of Ag atoms during bonding should be responsible for the overlap population of the Al-Al bond. Due to the substitution behavior of Ag for Ti, the replacement of Ag-C bond for Ti-C bond and formation of Ti-Ti and C-C anti-bonds reduced the structural stability, becoming the key factor in restricting the substitution ratio of Ag for Ti.

When Ag substituted for Al in the Ti_2_AlC, the bonding behavior of Ag was similar to that of Al, which produced the Ti-Ag bond adjacent to Ti atoms with the overlap population of 0.11 and bond length of 2.93873 Å (for the Ti_2_(Al_0.875_,Ag_0.125_)C). Nevertheless, the bonding behavior of Ag was similar to that of Ti (a transition element) when Ag substituted for Ti, giving rise to the Ag-Al and Ag-C bonds in adjacent positions. The overlap population and bond length of the Ag-Al bond were 0.33 and 2.69190 Å, respectively, while the overlap population and bond length of the Ag-C bond were 0.20 and 2.46208 Å, respectively (for the (Ti_0.9375_,Ag_0.0625_)_2_AlC). Compared to the Ti-Ag bond by Ag substituting for Al, the Ag-Al and Ag-C bonds driven by the substitution of Ag for Ti had a shorter bond length and stronger bond strength. That is, Ag would preferentially substitute for Ti in the Ti_2_AlC.

## 4. Conclusions

In the present work, the first-principles calculations were introduced to investigate the influence of Ag substitution for Al or Ti on structure and stability of the Ti_2_AlC MAX phase ceramic. The supercell parameter, formation and cohesive energies, density of state, and overlap population were evaluated during the substitution. The significant conclusions were drawn:

1. Ag could substitute for Al or Ti in Ti_2_AlC. With increasing the substitution ratio of Ag for Al, the supercell parameters of *a* and *b* of the Ti_2_(Al_1−*x*_,Ag*_x_*)C supercell showed almost no change, as well as the value of c increased slightly. For the volume of the supercell, it first increased and then decreased. The supercell parameters of *a* and *b* of the (Ti_1−*y*_,Ag*_y_*)_2_AlC supercell also exhibited no obvious change with increasing the substitution ratio of Ag for Ti. However, the value of *c* first increased and then decreased, while the volume of the supercell increased continuously. In general, for the compound containing more Ag, the symmetry of the hexagonal Ti_2_AlC crystal structure would be distorted. The formation and cohesive energies of the systems exhibited an increasing trend after the substitution of Ag for Al or Ti, suggesting the decline in structural stability of the Ti_2_AlC. Lastly, the substitution always occurred preferentially in one atomic layer.
2. The substitution limit of Ag for Al was calculated to be 12.5% for the Ti_2_(Al_1−*x*_,Ag*_x_*)C supercell without ruining lattice stability. During substitution, the bonding behavior of Ag atoms was similar to that of Al atoms. The original Al-Ti bond (overlap population: 0.30) was replaced by a weaker Ti-Ag bond (overlap population: 0.11), which should be responsible for the decline of the structural stability of the Ti_2_(Al_0.875_,Ag_0.125_)C. The replacement of Al-Ti bond by Ti-Ag bond generated excess electrons filling Ti *d* orbits, strengthening the Ti-Ti bond. The contribution of Ag 4*d* orbital electrons toward the DOS at *E_F_* coupled with the filling of Ti *d* orbital electrons improved electrical conductivity of the Ti_2_(Al_0.875_,Ag_0.125_)C significantly, compared to the original Ti_2_AlC.
3. When Ag substituted for Ti in the Ti_2_AlC, the bonding behavior of Ag atoms was similar to that of Ti atoms, which would give rise the Ag-Al and Ag-C bonds. In addition, the original Al-Ti bond became weaker as well as new Ti-Ti and C-C anti-bonds were generated (the anti-bonds became weaker with increasing the substation ratio) after substitution, being responsible for the decline of the structural stability. The compensation effect of Ag 4*d* orbital electrons toward electrical conductivity of the (Ti_1−*y*_,Ag*_y_*)_2_AlC was greater than the loss of Ti *d* orbital electrons driven by the substitution of Ag for Ti, improving the electrical conductivity. Through comparing the Ti-Ag bond for the substitution of Ag for Al with Ag-Al and Ag-C bonds for the substitution of Ag for Ti, it can be concluded that Ag atoms preferentially substituted for Ti atoms in the Ti_2_AlC.

## Figures and Tables

**Table 1:** Effect of substitution ratio of Ag for Al on lattice structure and stability of the Ti_2_(Al_1−*x*_,Ag_*x*_)C supercells.

Supercell	Supercell Parameter (Å)			Volume (Å^3^)	*E*_tot_ (eV)	*E*_f_ (eV)	*E*_coh_ (eV)
	*a*	*b*	*c*				
Ti_2_AlC	6.136	6.136	13.733	447.747	−27,380.253	−6.735	−0.767
Ti_2_(Al_0.875_,Ag_0.125_)C	6.134	6.134	13.833	450.763	−28,350.931	−6.667	−0.725
Ti_2_(Al_0.75_,Ag_0.25_)C	6.145	6.156	13.971	457.419	−29,320.755	−6.573	−0.657
Ti_2_(Al_0.625_,Ag_0.375_)C	6.118	6.119	13.971	452.983	−30,293.051	−6.555	−0.632
Ti_2_(Al_0.5_,Ag_0.5_)C	6.128	6.113	14.059	455.752	−31,263.752	−6.488	−0.624

**Table 2:** Atomic orbital charge populations of Ti_2_AlC and Ti_2_(Al_0.875_,Ag_0.125_)C supercells.

Supercell	Species	Ion	*s*	*p*	*d*	Total	Charge (e)
Ti_2_AlC	Ti		2.18	6.75	2.67	11.60	0.40
	Al		1.09	1.96	0.00	3.05	−0.05
	C		1.47	3.28	0.00	4.75	−0.75
Ti_2_(Al_0.875_,Ag_0.125_)C	Ti	2, 3, 6	2.18	6.73	2.63	11.54	0.46
	Al	1, 4, 6	1.09	1.94	0.00	3.03	−0.03
	C		1.47	3.28	0.00	4.76	−0.76
	Ag		0.76	1.02	9.70	11.48	−0.48

**Table 3:** Mulliken bond overlap populations of Ti_2_AlC and Ti_2_(Al_0.875_,Ag_0.125_)C supercells.

Supercell	Bond	Population	Length (Å)
Ti_2_AlC	C-Ti	0.39	2.10798
	Ti-Ti	0.04	2.89219
	Al-Ti	0.30	2.89517
Ti_2_(Al_0.875_,Ag_0.125_)C	C 004-Ti 002	0.41	2.10481
	Ti 002-Ti 008	0.09	2.89508
	Al 005-Ti 009	0.29	2.89347
	Ti 006-Ag 001	0.11	2.93873

**Table 4:** Effect of substitution ratio of Ag for Ti on lattice structure and stability of (Ti_1−*y*_, Ag*_y_*)_2_AlC supercells.

Supercell	Supercell Parameter (Å)			Volume (Å^3^)	*E*_tot_ (eV)	*E*_f_ (eV)	*E*_coh_ (eV)
	*a*	*b*	*c*				
Ti_2_AlC	6.136	6.136	13.733	447.747	−27,380.253	−6.735	−0.767
(Ti_0.9375_,Ag_0.0625_)_2_AlC	6.141	6.141	13.816	451.211	−26,801.127	−6.500	−0.652
(Ti_0.875_,Ag_0.125_)_2_AlC	6.169	6.163	13.761	452.959	−26,222.551	−6.344	−0.554

**Table 5:** Mulliken bond overlap populations of the Ti_2_AlC and (Ti_1__−*y*_,Ag*_y_*)_2_AlC supercells.

Supercell	Bond	Population	Length (Å)
Ti_2_AlC	C-Ti	0.39	2.10798
	Ti-Ti	0.04	2.89219
	Al-Ti	0.30	2.89517
(Ti_0.9375_,Ag_0.0625_)_2_AlC	C 003-Ti 012	0.44	2.08314
	Ti 001-Ti 010	0.01	2.87822
	Ti 008-Ti 014	−0.03	2.93187
	Al 003-Ti 006	0.24	2.94647
	Al 001-Ag 001	0.33	2.69190
	C 001-C 003	−0.09	2.94155
	C 001-Ag 001	0.20	2.46208
(Ti_0.875_,Ag_0.125_)_2_AlC	C 003-Ti 011	0.47	2.01204
	C 003-Ti 004	0.50	2.05016
	Ti 001-Ti 009	−0.02	2.85133
	Ti 007-Ti 009	−0.05	2.92157
	Al 002-Ti 001	0.24	2.93558
	Al 001-Al 007	0.23	2.96005
	C 001-Ag 001	0.21	2.44407
	C 007-Ag 001	0.24	2.42123
	Al 005-Ag 001	0.36	2.74509
	C 003-C 005	−0.09	2.92041

## Data Availability

The data presented in this study are available on request from the corresponding author.
